# Parental education associations with children’s body composition: mediation effects of energy balance-related behaviors within the ENERGY-project

**DOI:** 10.1186/1479-5868-10-80

**Published:** 2013-06-21

**Authors:** Juan M Fernández-Alvira, Saskia J te Velde, Ilse De Bourdeaudhuij, Elling Bere, Yannis Manios, Eva Kovacs, Natasa Jan, Johannes Brug, Luis A Moreno

**Affiliations:** 1 EMGO Institute for Health and Care Research and the Department of Epidemiology and Biostatistics, VU University Medical Center, Amsterdam, Netherlands; 2 GENUD (Growth, Exercise, Nutrition and Development) Research Group. Faculty of Health Sciences, University of Zaragoza, 50009 Zaragoza, Spain; 3 Department of Movement and Sport Sciences, Ghent University, Ghent, Belgium; 4 Department of Public Health, Sport and Nutrition, University of Agder, Kristiansand, Norway; 5 Department of Nutrition and Dietetics, Harokopio University, Athens, Greece; 6 Department of Paediatrics, Pecs University, Pecs, Hungary; 7 Slovenian Heart Foundation, Ljubljana, Slovenia

**Keywords:** Mediation analysis, Body composition, Parental education, Childhood obesity, Energy balance-related behaviors

## Abstract

**Background:**

It is well known that the prevalence of overweight and obesity is considerably higher among youth from lower socio-economic families, but there is little information about the role of some energy balance-related behaviors in the association between socio-economic status and childhood overweight and obesity. The objective of this paper was to assess the possible mediation role of energy balance-related behaviors in the association between parental education and children’s body composition.

**Methods:**

Data were obtained from the cross sectional study of the “EuropeaN Energy balance Research to prevent excessive weight Gain among Youth” (ENERGY) project. 2121 boys and 2516 girls aged 10 to 12 from Belgium, Greece, Hungary, the Netherlands, Norway, Slovenia and Spain were included in the analyses. Data were obtained via questionnaires assessing obesity related dietary, physical activity and sedentary behaviors and basic anthropometric objectively measured indicators (weight, height, waist circumference). The possible mediating effect of sugared drinks intake, breakfast consumption, active transportation to school, sports participation, TV viewing, computer use and sleep duration in the association between parental education and children’s body composition was explored via MacKinnon’s product-of-coefficients test in single and multiple mediation models. Two different body composition indicators were included in the models, namely Body Mass Index and waist circumference.

**Results:**

The association between parental education and children’s body composition was partially mediated by breakfast consumption, sports participation, TV viewing and computer use. Additionally, a suppression effect was found for sugared drinks intake. No mediation effect was found for active transportation and sleep duration. The significant mediators explained a higher proportion of the association between parental education and waist circumference compared to the association between parental education and BMI.

**Conclusions:**

Tailored overweight and obesity prevention strategies in low SES preadolescent populations should incorporate specific messages focusing on the importance of encouraging daily breakfast consumption, increasing sports participation and decreasing TV viewing and computer use. However, longitudinal research to support these findings is needed.

## Background

Overweight and obesity are important determinants of avoidable burden of disease [1]. Despite a leveling-off of obesity prevalence in some countries in the last years, childhood obesity still shows a high prevalence [2]. Recent cross-European data from the ENERGY project confirmed these high prevalence rates of overweight and obesity among schoolchildren [3]. It is also known that overweight and obesity track from childhood to adulthood [4,5]. Recent research and literature reviews show that, among schoolchildren, some specific energy balance-related behaviors (EBRBs) are associated with overweight and obesity prevalence and may be important for obesity prevention [6-9]. These behaviors comprise, among others, the intake of sugared drinks, skipping breakfast, screen viewing behavior (TV viewing and sedentary computer activities) and lack of regular physical activity, like active commuting to school, participation in sports and recreational physical activity. In addition, recent evidence suggests that sleeping habits may also be relevant for energy balance [10,11]. Despite the associations with overweight, many schoolchildren engage in these risk behaviors [3]. It is also well known that the prevalence of overweight and obesity is considerably higher among youth from lower socio-economic families [12-16].

There is little information about the role of those EBRBs in the association between indicators of socio-economic status and childhood overweight and obesity. Morgenstern et al. [17] have recently shown that the effect of socioeconomic status (SES) on overweight was partially mediated by media exposure in children and adolescents aged 10 to 17. Previous studies found that some behavioral factors (e.g. screen time, physical activity and dietary habits) may partially explain the association between SES and excess overweight prevalence, but did not include formal tests of mediation [17-19].

In the ENERGY cohort overweight and obesity were also more prevalent among children from parents with lower levels of education [3]. Moreover, the ENERGY project included data on the most important EBRBs. Therefore, the aim of this paper was to assess the possible mediation role of EBRBs in the association between parental education and children’s body composition. More specifically, the current study aims to assess: 1) the total associations of parental education with two different body composition outcomes: Body Mass Index (BMI) and waist circumference; 2) the associations of parental education with EBRBs (i.e. sugared drinks intake, breakfast consumption, active transportation to school, sports participation, TV viewing, computer use and usual sleep duration) as potential mediating variables; and 3) to assess the mediated pathways of EBRBs on BMI and waist circumference.

## Methods

### Study population and design

Data were obtained from the cross sectional study of the “EuropeaN Energy balance Research to prevent excessive weight Gain among Youth” (ENERGY) project [20]. This cross-sectional study was carried out between March and July 2010 in Belgium, Greece, Hungary, the Netherlands, Norway, Slovenia and Spain, among pupils in the final years of primary education (aged 10–12) [21]. The aim of the survey was to provide up to date information on the prevalence of overweight and obesity, on the most important energy balance related behaviors (EBRBs) and their social, cognitive and school environmental determinants. Based on previous cross-European studies [22], a minimum sample of 1000 schoolchildren per country and one parent/caretaker for each child were aimed for. The schools were randomly selected concerning the degree of urbanization of the different provinces and the socioeconomic status (SES) of the different areas within the selected provinces. Samples were national in Greece, Hungary, the Netherlands and Slovenia. In Spain, schools of the region of Aragón were selected; Belgium selected schools from Flanders and Norway selected schools from the southern regions of the country [21]. Descriptions of the rationale and design of the entire ENERGY project [20] and the procedures and methodology of the ENERGY school-based survey [21] are published elsewhere. All participants provided written informed consent prior their enrollment. The studies were approved by the corresponding local ethics committees in all participating countries. In Belgium the survey was approved by the Medical Ethics Committee of the University Hospital Ghent; In Greece the survey was approved by the Bioethics Committee of Harokopio University; In Hungary the survey was approved by the Scientific and Ethics Committee of Health Sciences Council; in the Netherlands the survey was approved by the Medical Ethics Committee of the VU University Medical Center; in Norway the survey was approved by the National Committees for Research Ethics in Norway; in Slovenia the survey was approved by the National Medical Ethics Committee of the Republic of Slovenia; and in Spain the survey was approved by Clinical Research Ethics Committee of the Government of Aragon. The project adhered to the Helsinki Declaration and the conventions of the Council of Europe on human rights and biomedicine.

### Measures

Measurements were conducted following standardized protocols. The children and the parents were asked to complete printed questionnaires assessing obesity related dietary, physical activity and sedentary behaviors, as well as potential determinants of engaging in these behaviors. The questionnaires and anthropometric measurements were completed during school hours. Test-retest reliability was tested by administrating the questionnaire twice with a one-week interval among 720 schoolchildren across the participating countries. In the paragraphs hereafter information on the intraclass correlation coefficients (ICC) is provided for the specific included items. Detailed information regarding the procedures, staff training and questionnaires development [21], and test-retest reliability and construct validity of the questionnaires are published elsewhere [23,24].

#### Anthropometric measurements

Body height, weight and waist circumference were measured by trained research assistants. Children were measured in light clothing without shoes. Body height was measured with a SECA Leicester Portable stadiometer (to the nearest 0.1 cm). Weight was measured with a calibrated electronic scale SECA 861 (to the nearest 0.1 kg), and waist circumference with a SECA 201 measuring band (to the nearest 0.1 cm). Two readings of each measurement were obtained. A third measurement was taken if the two readings differed more than 1%. Body Mass Index (BMI) and weight categories based on the International Obesity Task Force criteria (IOTF) [25] were calculated.

#### Parental educational level

As an indicator of socio-economic status, the parent respondents were asked to report their own level of education, as well as the level of education of the other parent/caregiver. Because educational systems differ considerably across Europe, years of formal education since preschool were used as the indicator for level of education. Answer categories were as follows: a) less than 7 years, b) 7–9 years, c) 10–11 years, d) 12–13 years and e) 14 years or more. After preliminary analyses of the distribution of the variable, it was concluded that to recode into low, medium and high parental education level was not possible due to the small sample size in the low category. Thus, parental education was categorized as being high (at least one parent more than 14 years of education) or low (both parents less than 14 years of education), which approximately distinguishes families with at least one caregiver who has completed medium or higher vocational, college or university training from other families.

#### Children’s energy balance-related behaviors

Sugared drinks consumption, breakfast consumption, active transportation, sports participation, TV viewing and computer use were reported by the children, while sleep duration was reported by the parents.

#### Dietary behaviors

Intakes of soft drinks and fruit juices were assessed with two food frequency questions. Children answered how many days per week they drank the beverage, answering on a seven-point scale from never to more than once every day. Afterwards they were asked to indicate how much they drank by ticking the number of glasses or small bottles (e.g. 250 ml), cans (i.e. 330 ml) and large bottles (i.e. 500 ml) for soft drinks, or glasses/small cartons (i.e. 250 ml) and regular cartons (330 ml) for fruit juices. The questionnaire included pictures of the serving sizes. These items showed moderate to good reliability (intraclass correlation coefficients (ICC_test-retest_) between 0.53 – 0.71). Mean intake in ml per day was calculated from these two questions. In addition, children were asked to fill in how much of the beverages they had consumed on the day before, following the same classification. For the purposes of this analysis, liters/day of sugared drinks (soft drinks + fruit juices) were taken into account. Breakfast consumption was assessed with two food frequency questions. Children answered how many days they usually eat breakfast during school days (ICC_test-retest_ = 0.73) and in the weekend (ICC_test-retest_ = 0.52). For the purposes of this analysis, total weekly days having breakfast were taken into account.

#### Physical activity behaviors

Active transportation to school was assessed by two questions about how many days per week the child cycled and/or walked to school (ICC_test-retest_ = 0.94 and 0.91), ranging from never to 5 days/week, and two questions on the duration of biking or walking to school, with 4 answer categories ranging from 1–5 minutes to more than 15 minutes (ICC_test-retest_ = 0.81 and 0.70). Total active transportation time per week was calculated by adding up total bike and walk times and multiplying the number of days with the mean time of the answering category times 2. Organized sports participation was assessed with specific questions about how many hours per week children participated in one or two sports (ICC_test-retest_ = 0.74 and 1.00). Based on the answers, average time of sports participation per week was calculated. Finally, minutes/week of active transportation and hours/week of sports participation were included in the analysis.

#### Sedentary behaviors

Screen time (i.e. TV and computer time) was assessed separately for weekdays and weekend days by two questions about time spent watching TV (including video and DVD) (ICC_test-retest_ = 0.67 and 0.68) and computer activities (ICC_test-retest_ = 0.67 and 0.67). Mean TV, computer and total screen time per day were calculated. For the analysis, total hours/week of TV viewing and total hours/week of computer use were taken into account.

#### Sleep duration

Child’s sleep duration reported by the parents included the number of hours the child sleeps per night on average, separately for weekdays (ICC_test-retest_ = 0.81) and weekend days (ICC_test-retest_ = 0.78). For the purpose of this paper, only weekdays (hours/day of sleep duration) were taken into account as sleep during weekdays is likely to be more representative of usual sleep duration, due to the more regular bed- and get-up routine [11].

### Statistical analysis

Means and standard deviations were calculated for the key variables, separately for boys and girls. A complete cases design was used; therefore only children having valid data for the variables included in the analysis were taken into account. Children not included in the final analysis had slightly lower BMI and were slightly older compared to the included sample (data not shown). Multilevel analysis intraclass correlation coefficients (ICCs) were calculated, in order to check whether a school level clustered design was needed. The obtained school ICCs were low (all <0.06), reflecting no clustering effect at school level. Therefore, the analyses were adjusted only for country level.

**Figure 1:**
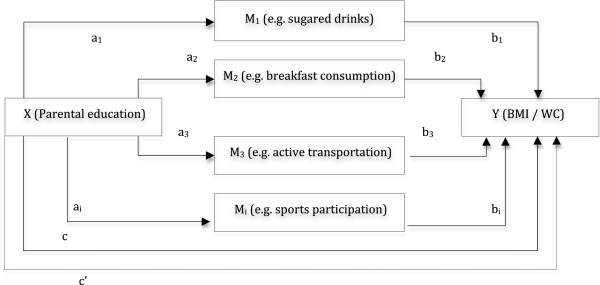
**Multiple mediator model.** X: predictor variable; Y: outcome variable; M_i_: mediator variable; a_i_: association between predictor (X) and potential mediator (M_i_); b_i_: association between potential mediator (M_i_) and outcome variable (Y); c: overall association between predictor variable (X) and outcome variable (Y); c’: direct effect (unmediated) of predictor variable (X) on outcome variable (Y).

Mediation analysis: To assess whether the associations between parental education and body composition outcomes were mediated by the EBRBs, the product-of-coefficients test proposed by MacKinnon was performed [26]. To qualify as a mediator, the presumed mediator has to be associated with the predictor variable and with the outcome variable [27]. To define the final regression models, the included EBRBs, as the presumed mediators, had to fulfill two requirements: (1) the parental education indicator (X) has to be associated with the mediator (M) (path a); and (2) the mediator has to be associated with the body composition outcome (Y) in a regression model adjusted for the predicting variable (X) (path b) (see Figure 1). The potential mediators meeting these criteria were included in the final multiple mediator models and mediation effects and mediated proportions were calculated. The product-of-coefficients method (a_i_*b_i_) was used to calculate the mediated effect. The total mediated effect was calculated as the sum of the individual mediated effects (Σ[a_i_*b_i_]). The mediated proportions were calculated as the mediation effect divided by the total effect (path c) ([a_i_*b_i_]/c) and (Σ[a_i_*b_i_]/c). Total effects were estimated by regression models without the potential mediators. Standard errors were calculated and used to construct the 95% confidence intervals (CI) for the direct and total effects. Bootstrap corrected CI were used for indirect, mediated, effects by means of the SPSS macro developed by Preacher and Hayes [28].

All regression models were adjusted for potential confounders (gender, age, country). Additionally, a potential effect modification of the total association by gender was assessed using an interaction term (gender*parental education). In case of significant interaction term, analysis would be stratified by sex. Analyses were conducted using IBM SPSS Statistics 19.

## Results

Table 1 describes the basic characteristics of the eligible children (2121 boys and 2516 girls). Differences on sugared drinks consumption, sports participation, TV viewing and computer use were found according to gender, while active transportation, breakfast consumption and sleep duration did not differ. Boys reported higher intake of sugared drinks, higher sports participation and higher TV and computer use compared to girls. There was also a higher proportion of overweight boys compared to girls, but no differences in obesity prevalence [3].

**Table 1:** Descriptive statistics of the study sample, by sex

**N = 4637**	**Boys**		**Girls**		
	**2121**		**2516**		
	**Mean**	**SD**	**Mean**	**SD**	**p-value**
Age (years)	11.6	0.73	11.6	0.73	0.089
Sugared drinks (liters/day)	0.63	0.63	0.52	0.58	<0.001
Breakfast (days/week)	6.02	1.80	5.95	1.81	0.179
Active transportation (min/week)	53.55	54.85	54.95	58.61	0.408
Sports participation (hours/week)	4.23	2.77	3.30	2.62	<0.001
TV viewing (hours/week)	13.43	6.93	12.49	6.83	<0.001
Computer use (hours/week)	10.52	7.06	7.71	6.16	<0.001
Sleep duration (hours/day)	9.05	0.86	9.07	0.89	0.388
	**n**	**%**	**n**	**%**	**p-value**
High parental education (n [%])	1449	68.3	1690	67.2	0.698
Normalweight (n [%])	1548	73.0	1975	78.5	<0.001
Overweight (n [%])	466	22.0	441	17.5	<0.001
Obese (n [%])	107	5.0	100	4.0	0.092

### Parental education and children’s body composition (c-path)

Analyses for effect modification by sex did not show significant results (all p-values > 0.324). Subsequently, all analyses below are presented for boys and girls together. The results showed that parental education was significantly inversely related with BMI (ß = −0.43; CI: -0.64, -0.23) and waist circumference (ß = −0.94; CI: -1.47, -0.41).

### Associations between parental education and energy balance-related behaviors (a-path)

As described in Tables 2 and Table 3 significant associations with parental education were found for sugared drinks, breakfast consumption, sports participation, TV viewing and computer use (all p < 0.001). Parental education was positively associated with sports participation and breakfast consumption, while sugared drinks, TV viewing and computer use were negatively associated with the parental education. Parental education was negatively associated with active transportation in the BMI model, but not in the waist circumference model.

**Table 2:** **Results from the mediation analyses in the association between parental education and BMI (kg/m**^**2**^**)**

**(n = 4636)**	**Parental education effect on mediator (path a, X → M)**			**Single mediator model Mediatior effect on BMI (path b, M → Y)**			**Multiple mediator model Mediator effect on BMI (path b, M → Y)**		
**Total association (path c) (95% CI): -0.43 (−0.64 to −0.23)**									
Mediator:	Beta	95% CI	p-value	Beta	95% CI	p-value	Beta	95% CI	p-value
Sugared drinks (l/day)	−0.14	−0.18 to −0.11	<0.001	−0.186	−0.346 to −0.025	0.233	−0.279	−0.443 to −0.116	<0.001
Breakfast (days/week)	0.26	0.15 to 0.37	<0.001	−0.211	−0.264 to −0.158	<0.001	−0.200	−0.253 to −0.147	<0.001
Active transportation (min/week)	−3.63	−6.99 to −0.28	0.034	0.001	−0.002 to 0.002	0.889			
Sports participation (hours/week)	0.50	0.33 to 0.66	<0.001	−0.062	−0.097 to −0.027	<0.001	−0.055	−0.090 to −0.021	0.002
TV viewing (hours/week)	−1.27	−1.67 to −0.81	<0.001	0.028	0.015 to 0.042	<0.001	0.024	0.009 to 0.038	0.002
Computer use (hours/week)	−1.19	−1.60 to −0.77	<0.001	0.020	0.005 to 0.034	0.006	0.010	−0.005 to 0.025	0.205
Sleep duration (h/day)	−0.04	−0.09 to 0.01	0.114	−0.309	−0.426 to −0.192	<0.001			
All significant mediators									
	Indirect effect (a*b, X → M → Y)			**Direct effect (path c′, X → Y**_**adj M**_**)**					Percentage mediated (a*b/c)		
Mediator:	Beta	95% CI		Beta	95% CI	p-value	%			
Sugared drinks (l/day)	0.03	0.03 to 0.05		−0.46	−0.66 to −0.26	<0.001	−6.1			
Breakfast (days/week)	−0.05	−0.08 to −0.03		−0.38	−0.58 to −0.18	<0.001	12.6			
Active transportation (min/week)	0.01	−0.01 to 0.01		−0.43	−0.64 to −0.23	<0.001	-			
Sports participation (hours/week)	−0.03	−0.05 to −0.02		−0.40	−0.61 to −0.20	<0.001	7.1			
TV viewing (hours/week)	−0.03	−0.06 to −0.02		−0.40	−0.60 to −0.20	<0.001	8.0			
Computer use (hours/week)	−0.02	−0.05 to −0.01		−0.41	−0.61 to −0.21	<0.001	5.4			
Sleep duration (h/day)	0.01	−0.01 to 0.03		−0.45	−0.65 to −0.24	<0.001	-			
All significant mediators	−0.08	−0.13 to −0.03		−0.35	−0.56 to −0.23	0.001	18.7			

For an explanation of a, b, c, c’, please see Figure 1.

All models were adjusted for age, gender and country.

**Table 3:** Results from the mediation analyses in the association between parental education and waist circumference (cm)

**(n = 4636)**	**Parental education effect on mediator (path a, X → M)**			**Single mediator model Mediator effect on WC (path b, M → Y)**			**Multiple mediator model Mediator effect on WC (path b, M → Y)**		
**Total association (path c) (95% CI): -0.94 (−1.47 to −0.41)**									
Mediator:	Beta	95% CI	p-value	Beta	95% CI	p-value	Beta	95% CI	p-value
Sugared drinks (l/day)	−0.14	−0.18 to −0.11	<0.001	−0.085	−0.504 to 0.335	0.693			
Breakfast (days/week)	0.26	0.15 to 0.37	<0.001	−0.484	−0.622 to −0.346	<0.001	−0.444	−0.582 to −0.305	<0.001
Active transportation (min/week)	−3.74	−7.09 to 0.39	0.029	−0.002	−0.006 to 0.003	0.489			
Sports participation (hours/week)	0.50	0.34 to 0.67	<0.001	−0.231	−0.323 to −0.139	<0.001	−0.219	−0.311 to −0.128	<0.001
TV viewing (hours/week)	−1.26	−1.69 to −0.83	<0.001	0.089	0.053 to 0.124	<0.001	0.068	0.029 to 0.107	0.001
Computer use (hours/week)	−1.19	−1.61 to −0.78	<0.001	0.058	0.021 to 0.095	0.002	0.019	−0.021 to 0.059	0.342
Sleep duration (h/day)	−0.04	−0.09 to 0.01	0.105	−0.676	−0.981 to −0.370	<0.001			
All significant mediators									
	Indirect effect (a*b, X → M → Y)			**Direct effect (path c′, X → Y**_**adj M**_**)**			Percentage mediated (a*b/c)		
Mediator:	Beta	95% CI		Beta	95% CI	p-value			%	
Sugared drinks (l/day)	0.01	−0.05 to 0.07		−0.95	−1.48 to −0.42	0.001			-	
Breakfast (days/week)	−0.13	−0.20 to −0.06		−0.81	−1.34 to −0.28	0.003			13.5	
Active transportation (min/week)	0.01	−0.01 to 0.04		−0.96	−1.49 to −0.43	0.001			-	
Sports participation (hours/week)	−0.12	−0.18 to −0.06		−0.96	−1.49 to −0.43	0.002			12.4	
TV viewing (hours/week)	−0.11	−0.19 to −0.06		−0.83	−0.36 to −0.30	0.002			11.9	
Computer use (hours/week)	−0.07	−0.14 to −0.03		−0.87	−1.40 to −0.34	0.001			7.4	
Sleep duration (h/day)	0.03	−0.01 to 0.08		−0.97	−1.49 to −0.44	<0.001			-	
All significant mediators	−0.34	−0.46 to −0.23		−0.60	−1.13 to −0.07	0.026			35.8	

For an explanation of a, b, c, c’, please see Figure 1.

All models were adjusted for age, gender and country.

### Associations between energy balance-related behaviors and children’s body composition (path-b)

TV viewing and computer use were positively associated with both BMI and waist circumference, while sports participation, breakfast consumption and sleep duration were negatively associated with both BMI and waist circumference (all p < 0.01). Sugared drinks consumption was also negatively associated with BMI (p < 0.05), but not with waist circumference. These variables were selected to be included in the final multiple mediator models, as shown in the third column of Table 2 and Table 3.

### Mediation effects (a*b)

The fourth and sixth columns of Tables 2 and Table 3 show the estimated mediation effects and the proportion mediated. Indirect effects (see Figure 1) were statistically significant for breakfast consumption, sports participation, TV viewing and computer use in both models, and for sugared drinks in the BMI model. The mediated proportions varied, with the highest proportion for breakfast consumption (12.6%-13.5%) and the smallest for computer use (5.4%-7.4%). The total proportions of the overall effect mediated by all the mediators were 18.7% for the BMI model and 35.8% for the waist circumference model.

Sugared drinks consumption had a suppressive effect on the relationship between parental education and BMI (−6.1%), due to opposite directions of the direct and indirect associations.

### Direct association (path-c’)

As can be seen in Tables 2 and Table 3, there were significant direct associations between parental education and BMI and waist circumference, after inclusion of the presumed mediators in the model.

## Discussion

The present study aimed to explore the mechanisms through which parental education differences are associated with children’s body composition. Current results first confirmed that parental educational level was inversely associated with children’s BMI and waist circumference. Next, the results showed that the association between parental education and children’s body composition was partially mediated by certain EBRBs, namely breakfast consumption, sports participation, TV viewing and computer use. Additionally, a suppression effect was found for sugared drinks intake. No mediation effect was found for active transportation and sleep duration.

Although previous studies looked at the separate relationships and not at the mediation effect, the results are in line with previous reports in which higher parental education was associated with lower childhood overweight indices [29,30]. The associations between parental education and the EBRBs are also in line with available literature showing a negative association between several SES-indicators (including parental education) and breakfast skipping [31], sedentary habits [32], sugared drinks intake [33] and active transportation [34], and a positive association between SES and physical activity [32].

The current results on the association between the different EBRBs and the body composition outcomes are also in agreement with previous reports. Physical activity level, TV viewing, sugared drinks intake, breakfast consumption and sleep duration have been clearly associated with childhood overweight, while computer use was found to be related only in some studies [9,35-38]. Previous reports on the association between active transportation and overweight did not show a consistent relationship [18,39].

Although sleep duration has been associated negatively with childhood overweight – which was also confirmed in the present data-, the current analysis did not show a mediation effect of sleep duration, due to a non-significant association between parental education and sleep duration. In contrast, parental education was associated with active transportation, but active transportation was not associated with body composition indices. Recent reports showed positive associations between parental education and cycling to school while negative associations between cycling to school and overweight have been reported [40,41]. Therefore, the single mediation analysis was also repeated for the two active transportation categories separately, in order to look at the specific trends. We found a negative association between parental education and walking to school but no association between parental education and cycling to school. In both cases no association with body composition indices was found (data not shown).

A suppression effect was found for sugared drinks intake. This effect could be due to a lower intake of sugared drinks in overweight children, or a higher impact of underreporting in this group [42-44]. It is also noteworthy that sugared drinks consumption was a suppressor only in the BMI model, but not in the waist circumference model.

The analyses included two different body composition indicators, namely BMI and waist circumference. The significant mediators explained 36% of the relationship between parental education and waist circumference, while only 19% of the relationship between parental education and BMI was explained. This finding could reflect the differences between BMI and waist circumference as measures of childhood adiposity. Waist circumference has been observed in cross-sectional studies to be a good abdominal fat estimate [45], a better predictor of cardiovascular disease risk factors in childhood [46,47], and to predict cardio-metabolic health risk in adults beyond that explained by BMI [48,49].

To our knowledge, this is one of the first studies trying to disentangle the complex interaction between parental education, several EBRBs and children’s body composition by applying mediation analysis. The proportion of the associations explained by the included mediators in both models was moderate, remaining the direct associations (c-path) still significant after adjusting for the mediators (c’-path). These results suggest that other variables not included in the analyses may play a mediating role in the relationship between parental education and children’s body composition. Future analyses including other potential mediators, not only behavioral but also environmental factors, like availability or accessibility, could enhance the knowledge about the complex relationship between parental education and children’s body composition.

It has to be kept in mind that this study is subject to some limitations. First, this concerns a cross-sectional study providing evidence for associations but not causation. Further, data on dietary, physical activity and sedentary behaviors were based on self-reports, and thus possibly biased. However, the measures showed good test-retest reliability and construct validity [23,24]. When considering sedentary behaviors it is also important to note that some sedentary activities, like reading or studying were not included in the present study. Is therefore possible that questionnaires did not reflect the real, total time spent in sedentary behaviors. Parental education was reported taking into account both parents, and therefore we were not able to assess the differential influence of paternal and maternal educational levels on children’s EBRBs. Although the use of central adiposity indicators like waist circumference is useful as a good predictor of future health problems, it could be helpful to control for the maturation level when assessing pre- and adolescent population [50]. Unfortunately, no information on maturation level was collected in the ENERGY cross-sectional study. Finally, the differences between selected participants and those not included in the analysis may influence the generalizability of the results. However, differences were small and, although significant, probably not relevant.

Strengths of the present study include the large multinational sample from different regions across Europe, the available measured weight, height and waist circumference and the standardized data collection protocol across the different centers.

## Conclusions

The association between parental education and children’s body composition was partially mediated by breakfast consumption, sports participation, TV viewing and computer use. The significant mediators explained a higher proportion of the association between parental education and waist circumference compared to the association between parental education and BMI. Tailored overweight and obesity prevention strategies in low SES preadolescent populations should develop specific messages focusing on the importance of encouraging daily breakfast consumption, increasing sports participation and decreasing TV viewing and computer use.

## Competing interests

The authors declare that they have no competing interests.

## Author’s contributions

JMFA carried out the statistical analysis and drafted the manuscript. SJTV supervised the statistical analysis and design. IDB, EB, YM, EK, NJ and LAM collected the data in their countries or supervised data collection. JB is the coordinator of the ENERGY-project and helped to draft the manuscript. LAM supervised the statistical analysis and helped to draft the manuscript. All co-authors read and approved the final version of the manuscript.
